# First clinical application of cord blood mesenchymal stromal cells in children with multi-drug resistant nephrotic syndrome

**DOI:** 10.1186/s13287-022-03112-7

**Published:** 2022-08-19

**Authors:** William Morello, Silvia Budelli, Daniel Ari Bernstein, Tiziana Montemurro, Elisa Montelatici, Cristiana Lavazza, Luciana Ghio, Alberto Edefonti, Licia Peruzzi, Daniela Molino, Elisa Benetti, Bruno Gianoglio, Florian Mehmeti, Laura Catenacci, Jessica Rotella, Chiara Tamburello, Antonia Moretta, Lorenza Lazzari, Rosaria Giordano, Daniele Prati, Giovanni Montini

**Affiliations:** 1 Pediatric Nephrology, Dialysis and Transplant Unit, Fondazione IRCCS Ca’ Granda Ospedale Maggiore Policlinico, Via della Commenda 9, 20122 Milan, Italy; 2 Laboratory of Regenerative Medicine, Cell Factory, Department of Transfusion Medicine and Hematology, Fondazione IRCCS Ca’ Granda Ospedale Maggiore Policlinico, Via Francesco Sforza 35, 20122 Milan, Italy; 3 Pediatric Nephrology Unit, Regina Margherita Children’s Hospital, AOU Città della Salute e della Scienza di Torino, Turin, Italy; 4 Pediatric Nephrology and Dialysis Unit, Santobono Children’s Hospital, Naples, Italy; 5 Department of Women’s and Children’s Health, University Hospital of Padua, Padua, Italy; 6 Pediatric Hematology Oncology and Cell Factory, Department of Maternal and Children’s Health, Foundation IRCCS Policlinico San Matteo, Pavia, Italy; 7 Department of Clinical Sciences and Community Health, University of Milan, Milan, Italy

**Keywords:** Idiopathic nephrotic syndrome, Mesenchymal stromal cells, Multi-drug resistant nephrotic syndrome, Children, Advanced therapy medical products, Cord-blood-derived mesenchymal stromal cells

## Abstract

**Background and objectives:**

Children with multi-drug resistant idiopathic nephrotic syndrome (MDR-INS) usually progress to end-stage kidney disease with a consistent risk of disease recurrence after transplantation. New therapeutic options are needed for these patients. Mesenchymal stromal cells (MSCs) are multipotential non-hematopoietic cells with several immunomodulatory properties and growing clinical applications. Cord blood-derived MSC have peculiar anti-inflammatory and immunosuppressive properties. We aimed at assessing safety and efficacy of cord-blood-derived MSCs (CB-MSCs) in children with MDR-INS.

**Design, setting, participants:**

Prospective, open-label, single arm phase I–II pilot study. Pediatric patients with MDR-INS, resistant to at least two lines of therapy, were enrolled. Allogenic CB-MSCs were administered intravenously on days 0, 14, and 21 at a dose of 1.5 × 10^6^ cells/kg. Patients were followed for at least 12 months. The primary outcomes were safety and toxicity. The secondary outcome was remission at 12 months evaluated by urinary protein/urinary creatinine ratio (uPr/uCr). Circulating regulatory T cells (Tregs) were monitored.

**Results:**

Eleven pediatric patients with MDR-INS (10 females, median age 13 years) resistant to a median of 3 previous lines of therapy were enrolled. All patients completed the CB-MSC infusion schedule. No patient experienced any infusion-related adverse event or toxicity. Nine patients were assessable for efficacy. At the 12 months follow-up after the treatment, the median uPr/uCr did not change significantly from baseline (8.13 vs. 9.07; *p* = 0.98), while 3 patients were in partial or complete remission. A lower baseline uPr/uCr was a predictor of remission (2.55 vs. 8.74; *p* = 0.0238). Tregs count was not associated with CB-MSCs therapy.

**Conclusions:**

CB-MSCs are safe and may have a role in the immunosuppressive therapy of pediatric patients with MDR-INS. This preliminary experience paves the way toward further phase II studies addressing MSC efficacy in immune-mediated kidney diseases.

**Supplementary Information:**

The online version contains supplementary material available at 10.1186/s13287-022-03112-7.

## Introduction

Idiopathic Nephrotic Syndrome (INS) is the most common glomerular disease in children, and it is characterized by the clinical triad of nephrotic-range proteinuria, hypoalbuminemia and edema [1]. The pathogenesis has not been completely clarified, but it is thought to involve immunological processes and a yet unknown circulating factor [2]. Approximately 15% of children are resistant to the initial treatment with steroids and are defined as steroid-resistant (SRNS) [3]. Multi-drug resistant (MDR) patients, failing to achieve remission with second-line immunosuppressive therapies, are at high risk of end-stage kidney disease (ESKD) with a 55% chance of disease recurrence after kidney transplantation [4]. New therapeutic options, with novel mechanisms of action, are extremely needed for MDR patients.

Mesenchymal stromal cells (MSCs) are multipotent cells defined by their capacity to adhere to the plastic in standard culture conditions, to differentiate into bone, adipose tissue and cartilage and to highly express (> 95%) mesenchymal antigens (CD105, CD73 and CD90) while being negative (< 2%) to hematopoietic antigens (CD45, CD34. CD14 or CD11b, CD79 alfa or CD19) and HLA-DR [5–7]. They can be isolated from several adult and fetal tissues, including bone marrow and umbilical cord blood (CB) [8, 9]. MSCs are well-known to contribute to tissue repair in chondrogenic and osteogenic damaged tissues [10] and reduce apoptosis in damaged cells supporting tissue regeneration [11–13], but the most clinically relevant therapeutic effect relies on their immunomodulatory properties. Indeed, MSCs interact with each cellular component of the immune system. MSCs (1) inhibit the antigen-presenting and phagocytic capacity of monocytes/macrophages while inducing their expression of interleukin (IL)-10 and programmed cell death ligand 1 [14]; (2) affect the maturation of dendritic cells and their secretion of pro-inflammatory cytokines [15]; (3) inhibit the proliferation [16] and pro-inflammatory properties of CD4^+^T helper (Th)1 and Th17 cells [17], while stimulating the proliferation of regulatory T cells [18]; (4) impair the expansion, cytokine secretion, and cytotoxic activity of CD8^+^T cells [16]; (5) inhibit B-cell differentiation, proliferation, and antibody secretion and enhance the generation of IL-10-producing cell subsets [19]. MSC therapy has a proven therapeutic efficacy in the setting of graft versus host disease (GvHD) [20, 21]. Beyond GvHD, MSCs were able to produce immunosuppression in preclinical models and experimental clinical setting of immunological disorders such as arthritis, systemic lupus erythematosus, and multiple sclerosis [8].

On the basis of the immunomodulatory and anti-apoptotic effects of MSCs, we aimed at assessing the safety and efficacy of cord blood MSCs (CB-MSCs) in children with MDR-INS.

## Materials and methods

We performed a monocentric, prospective, open-label, single arm phase I-II pilot study with allogenic CB-MSCs in pediatric patients with MDR-INS between April 2015 and April 2019.

### Population

Inclusion criteria were as follows: (1) clinical diagnosis of SRNS; (2) failure to achieve remission with at least one additional line of immunosuppressive therapy, after steroids; (3) age ≤ 18 years; (4) minimal change disease or focal segmental glomerulosclerosis at kidney biopsy; (5) estimated glomerular filtration rate (eGFR) ≥ 30 ml/min/1.73 m^2^ based on the Schwartz formula [22]; (6) no documented mutations in genes associated with genetic INS. Patients with ESKD and/or a known genetic disease were excluded for the low likelihood to benefit from the treatment. The study was approved by the local ethics committee and by the national competent authorities for clinical trial authorization (Istituto Superiore di Sanità and Agenzia Italiana del Farmaco [AIFA]). EudraCT number 2011-001387-21. A written informed consent was obtained from parents or legal guardians before the enrollment of children. All data were anonymized and de-identified prior to the analysis.

### CB-MSC production and batch selection

CB was provided by the Milano Cord Blood bank located in Fondazione IRCCS Ca’ Granda Ospedale Maggiore Policlinico in Milano. CB units were collected at birth after obtaining written informed donor consent from the mothers. Samples from the mothers at birth were tested negative for HBsAg, anti-HCV, anti-HIV 1 e 2, TPHA/VDRL, HBV DNA, HCV RNA, HIV RNA and for any additional marker following the Italian rules. The collection and all the other subsequent procedures, including transportation to the processing laboratory, were performed in accordance with the FACT-JACIE International Standards for Hematopoietic Cellular Therapy Product Collection, Processing and Administration, current edition {http://www.factwebsite.org/}. CB**-**MSC Good Manufacturing Practices (GMP) production was performed at the Cell Factory of Fondazione IRCCS Ca’ Granda Ospedale Maggiore Policlinico in Milan, Italy. The facility is a fully controlled plant for ATMP manufacturing; its characteristics, as well as the main quality assurance procedures, were previously described [23–26]. All manufacturing procedures were performed in a class A environment (Class II Type A2 Biological Safety Cabinet) with a class B surrounding environment. Microbial contamination was monitored using settle plates and volumetric active air sampling, and surfaces and operators were sampled with contact plates. In-continuous airborne particle monitoring was performed by means of automatic particle counters in the class A environment and during the critical steps in the class B environment. The procedures for manufacturing were developed by the authors (manuscript in process). Briefly, whole cord blood was centrifuged at 1900 rpm for 15 min and the buffy coat re-suspended at a concentration of 50,000 cells/cm^2^ in cell culture chambers in alphaMEM (Macopharma) supplemented with 20% qualified fetal bovine serum (FBS; Thermo Fisher Scientific). Cultures were maintained at 37 °C in a humidified atmosphere containing 5% CO2. After 48–72 h, non-adherent cells were removed and the fresh medium was added. The culture medium was changed every 3 days. At day + 10, the cells were harvested using TrypLE Select 1X (Thermo Fisher Scientific), and sub-cultured at a concentration of 1000–4000 cells/cm^2^. A subsequent medium change was performed at day + 13 and day + 20. At day + 17 and day + 24, the culture was trypsinized to detach adherent cells: CBMSCs were harvested and re-seeded at a concentration of 1000–4000 cells/cm^2^ until passage 5. Cells were cryopreserved in 20 mL of a solution composed of normal saline, human albumin (10% vol:vol), and DMSO (10% vol:vol) in specific cryobags (CryoMACS Freezing bag 50, Miltenyi Biotec), using a controlled-rate freezing system. The MSCs phenotype was determined by multicolor flow cytometry according to ISCT minimal criteria [6] with a panel of monoclonal antibodies directed against the surface molecules CD45, CD73, CD90, CD105 (all from BD Biosciences, San Jose, CA, USA). The cell viability was assayed by flow cytometry by performing propidium iodide (PI, BD Biosciences, San Jose, CA, USA) staining 0.1 × 10^6^ MSCs cells also stained for CD45 and CD90. Samples were assayed with a FACSCanto II cytometer (BD, Franklin Lakes, NJ, USA); data were analyzed using Diva 8.0 software (BD). The final products to be released had to be sterile, non-pyrogenic (endotoxin level < 0.25 EU/mL), must had a purity of ≥ 90% CD45-/CD90 + /CD105 + with CD45 + contaminants ≤ 2%; viability > 80% and a normal karyotype (46, XX or 46,XY). All the methods for quality controls have been validated as already published [24]. CB-MSCs batches were selected by an in-vitro alloreactivity test performed at the *Pediatric Hematology Oncology Lab, Fondazione IRCCS Policlinico S. Matteo, Pavia.* Patients’ peripheral blood mononuclear cells (PBMCs) were isolated by density-gradient centrifugation (Cell Separation Media, CEDERLANE; Burlington, CA) and plated overnight at 1 × 10^6^/ml concentration in RPMI-1640 (Euroclone; Milano, IT) at 37 °C in humidified 7% CO^2^ atmosphere. The lytic activity of PBMCs against chromium (^51^Cr)-labeled MSCs from different batches was measured in a 5 h-chromium release assay. MSCs that were resistant to the lytic activity were selected for the procedure. In addition, the presence of anti-HLA antibodies specifically directed against the HLA class I molecules of the CB-MSC was evaluated.

### Treatment schedule

CB-MSCs were administered as three intravenous infusions on days 0, 14, and 21 at a dose of 1.5 × 10^6^ cells/kg of body weight, over a period of 10 min in an outpatient setting. Treatment schedule and dosage were based on previous clinical experience with MSCs [8]. Patients were pre-medicated with paracetamol 10 mg/kg and Cetirizine 0.5 mg/kg 20 min before CB-MSCs administration, as already done in other MSC clinical trial to prevent transient febrile and limited urticarial reactions to the cells and/or their excipients. The ongoing therapy was not modified at the time of the infusions and no modifications of the immunosuppressive therapy were allowed for at least one year after the treatment.

### Outcomes

The primary outcomes were safety and toxicity [27] of CB-MSC in children with MDR-INS. Secondary outcomes were: (1) Response to therapy at 12 months, classified as partial remission if the urinary protein/urinary creatinine ratio (uPr/uCr) was between 0.2 and 2 mg/mg or complete remission if uPr/uCr was < 0.2 mg/mg. Patients were categorized as ‘responders’ if they achieved a partial or complete remission, otherwise they were considered as ‘non-responders’. (2) Reduction in ongoing immunosuppressive and antiproteinuric agents.

### Follow-up

Study visits were planned at screening, at each CB-MSCs administration, 2 weeks after the last infusion (follow-up 1, FUP1), and then every 6 weeks. Additional visits occurred whenever deemed clinically appropriate or in case of complications or possible relapses. Study coordinators maintained weekly contacts with families, to identify and treat potential adverse events. Data were collected until 12 months after enrollment.

During visits, patients underwent a physical examination and laboratory evaluations, including complete blood count, proteinuria, serum proteins, cholesterol, kidney and liver function tests. Kidney function was monitored using estimated GFR (Schwartz formula) [22]. We defined kidney function as ‘stable’ as long as the CKD category remained the same throughout the follow-up period.

### Regulatory T cells (Tregs) monitoring

At each study visit a blood sample was taken for Treg quantification. Tregs were isolated and quantified at the Pediatric Hematology Oncology Lab, Fondazione IRCCS Policlinico S. Matteo, Pavia. For surface staining PBMCs, collected after overnight incubation in RPMI medium, were first incubated with anti-human CD4-Pe-Cy7, anti-human CD25-APC, anti-human CD127-FITC (eBioscience TM, CA). After cell fixation and membrane permeabilization, intracellular staining with anti-Foxp3-PE (Anti-human Foxp3 staining set PE kit; eBioscienceTM, Carlsbad, CA) was performed. Tregs were measured by CD4 T cells gating on the lymphocyte region (forward/sideward scatter), and the frequency of CD25 + CD127^neg^ Foxp3 + cells was calculated as a proportion of CD4 + T cells on an 8-color flow cytometer (FACS Navios, Beckman Coulter).

### Serum cytokine evaluation

To evaluate the inflammatory environment before MSC administration, levels of interleukin (IL)-6, IL-8, tumor necrosis factor-α (TNF-α), IL-1β, IL-10 and interferon-γ (IFN-γ) were measured in patient’s peripheral blood (sera) at baseline, before the first MSC administration. Measurements were made using a high-sensitivity planar enzyme-linked immunosorbent assay with a chemiluminescent substrate (CorPlex Human Cytokine Panel 1, Quanterix, MA). The samples were centrifuged and stored at − 80 °C. The values were expressed as median (and range) and they were compared with reference value in normal subjects as reported by the literature [28].

### Statistical analysis

Considering the rarity of the disease and the pilot nature of the study, for sample size calculation we used the formula proposed by Viechtbauer et al. [29]. With an expected response rate of 25% and a confidence interval of 95%, we defined the sample size as 10, further increased by 10% to address possible drop-outs. The Mann–Whitney test was used to compare the data of patients who achieved remission and patients who did not. Data analysis was performed by a trained biostatistician.

## Results

We enrolled 11 pediatric patients with MDR-INS, ten females and one male, with a median age of 13 years (range: 7–18). At the time of enrollment, the median duration of disease was 32 months (range: 7–142), with a median of 3 (range: 2–6) previous lines of therapy, including steroids, CNIs, Mycophenolate Mofetil (MMF), Rituximab (RTX) and Ofatumumab (OFA). The median uPr/uCr was 8.13 mg/mg (range: 2.43–18.26) at study entry. The characteristics of the enrolled population are summarized in Table 1 and Additional file 1: Table S1. All included patients were under immunosuppressive therapy with at least one agent: 5 patients with steroids, 5 patients with cyclosporin (CsA), 4 patients with MMF and 3 patients with tacrolimus (TAC) (Table 2).

**Table 1:** Baseline clinical characteristics of enrolled patients

Characteristic	Median (range)
Age (years)	13 (7–18)
Gender	10 F: 1 M
Disease duration (months)	32 (7–142)
Proteinuria (uPr/uCr mg/mg)	8.13 (2.43–18.26)
Previous lines of therapy	3 (2–6)
eGFR (mL/min/1.73m^2^)	113.9 (55.7–139)
Serum creatinine (mg/dL)	0.74 (0.28–1.79)
Serum total protein (g/dL)	4.5 (3.6–5.6)
Serum albumin (g/dL)	2.6 (1.5–3.5)
Total cholesterol (mg/dL)	347 (167–684)
Triglycerides (mg/dL)	232 (110–552)
Serum cytokine level (pg/mL)	
IFN-gamma	0.065 (0.03–2.612)
IL-6	1.304 (0.699–5.537)
IL-8	17.8 (13.9–50.2)
IL-10	1.1 (0.5–2.2)
TNF-alfa	6.485 (2.765–13.159)
IL-1 beta	0.121 (0.091–0.574)

**Table 2:** Undergoing immunosuppressive therapy at enrollment

Patient	Therapy
Patient #1	PDN, CsA
Patient #3	PDN, CsA, MMF
Patient #5	CsA
Patient #6	TAC, MMF
Patient #7	PDN, CsA, MMF
Patient #8	TAC
Patient #9	PDN, CsA, MMF
Patient #10	TAC
Patient #11	PDN

*PDN* prednisone, *CsA* cyclosporine-A, *MMF* mycophenolate mofetil, *TAC* tacrolimus

As per inclusion criteria, at the time of enrollment, no patients had a documented mutation in genes involved in SRNS pathogenesis. Two patients (patient 2 and patient 4), both females, were later discovered to have a genetic form of SRNS in an additional genetic screening with Next Generation Sequencing, performed after the study. Patient 2 is a compound heterozygote for mutations in the *NPHS2* gene: c.[686G > A(;)946C > T] p.[Arg229Gln(;)Pro316Ser] exons 5 and 8, and heterozygote for the *NPHP4* gene: c.2542C > T p.Arg848Trp exon 19. Patient 4 is a carrier of a mutation in the *WT1* gene: c.1447 + 5G > A. Consequently, they were both withdrawn from the efficacy analysis, but their data were included in the safety analysis.

As per protocol, all patients received three CB-MSC intravenous infusions on days 0, 14, and 21. The median dose was 1.5 × 10^6^ cells/kg (1.1–2) equal to 70 × 10^6^ total cells (35–120) per administration. The median purity was 99.2% (92–99.9) with a median CD45 + contaminants of 0.2% (0–0.5) and a viability of 90.75% (84.9–95.1). All the products were sterile, mycoplasma-free and with a level of endotoxins below the limit of 0.25 EU/mL. CB-MSC dose and quality as single patient’s and overall data are reported in Additional file 1: Table S2 and Table S3.

All patients completed the 12 months’ follow-up and additional information was available for 10 patients, with a total median follow-up of 45 (range: 12–78) months.

### Primary outcome

All patients completed the study, and data were available for safety analysis in all 11 treated patients. The infusions were well tolerated, without any infusion-related adverse event. No patient experienced any grade toxicity, no one experienced allergic reactions, respiratory distress, fever or liver impairment. No patient had a deterioration of kidney function that could be ascribed to the therapy.

### Secondary outcomes

As regards the efficacy, the median value of proteinuria of all 9 assessable patients did not change significantly after treatment. The median baseline proteinuria was 8.13 (range: 2.43–18.26), 7.21 (range: 1.23–16.52) at the first follow-up (after the third infusion), and 9.07 (range: 0.23–19.31) at the end of follow-up period (12 months) (*p* = 0.98).

Nonetheless, after CB-MSCs treatment, three out of the nine patients with no genetic mutations met the criteria for partial or complete remission with a reduction in proteinuria below the nephrotic range associated with an improvement of serum proteins and serum cholesterol. In two cases (patients 3 and 9) this reduction was persistent, while in the third one (patient 6) it was temporary. In particular, patients 3 and 9 reached a partial remission at 12 months with uPr/uCr of 0.35 and 0.94 mg/mg, respectively. Patient 3 achieved a complete remission (uPr/uCr 0.15 mg/mg) 16 months after the first infusion and remains in remission at the last available follow-up. In this case it was also possible to progressively reduce and eventually discontinue the ongoing immunosuppressive treatment and renin–angiotensin–aldosterone system inhibitors. Patient 9 displayed a slow but progressive reduction in proteinuria, with a uPr/uCr of 0.44 mg/mg at the last follow-up, 26 months after CB-MSCs therapy. Patient 6, instead, achieved partial remission 7 months after treatment (uPr/uCr 1.12 mg/mg), with a nadir of proteinuria (uPr/uCr 0.23 mg/mg) at 11 months. Her remission lasted for a total of 10 months, with relapse occurring without any apparent triggering event.

As expected, MSC therapy had no effect on both patients with genetic SRNS, which progressed to ESKD according to the natural evolution of their disease.

Patients and disease characteristics were compared between responders and non-responders in order to identify the predictors of response to CB-MSCs (Table 3). The median baseline proteinuria of the patients who achieved remission was significantly lower than those who did not (uPr/uCr 2.55 mg/mg vs. 8.74 mg/mg; *p* = 0.0238). eGFR (223.7 vs. 95.95, *p* = 0.0238), serum creatinine (0.30 vs. 1.08, *p* = 0.0238), serum total proteins (5.40 vs. 4.00, *p* = 0.0419), serum albumin (3.40 vs. 2.18, *p* = 0.0238), serum cholesterol (226.0 vs. 444.50, *p* = 0.0357) and triglycerides (143.0 vs. 383.5, *p* = 0.0238) were significantly different between those groups as well. No significant differences were observed with regards to age, disease duration, lines of therapy prior to the study and ongoing therapy at the time of infusions.

**Table 3:** Patients and disease characteristics in responders and non-responders

	Responders (n = 3)	Non-responders (n = 6)	*p*-value
uPr/uCr (mg/mg)	2.55	8.74	0.0238
eGFR (mL/min/1.73 m^2^)	223.7	95.95	0.0238
Serum Creatinine (mg/dL)	0.30	1.08	0.0238
Serem proteins (g/dL)	5.40	4.00	0.0238
Albuminemia (g/dL)	3.40	2.18	0.0238
Cholesterolemia (mg/dL)	226.00	444.50	0.0357
Triglycerides (mg/dL)	143.00	383.5	0.0238
Age (years)	7	14	0.1486
Disease Duration (months)	40	30	0.5476
Previous Lines of Threapy (#)	4	3.5	1.0000
Ongoing Therapy (#)	3	1	0.0977

**Fig. 1:**
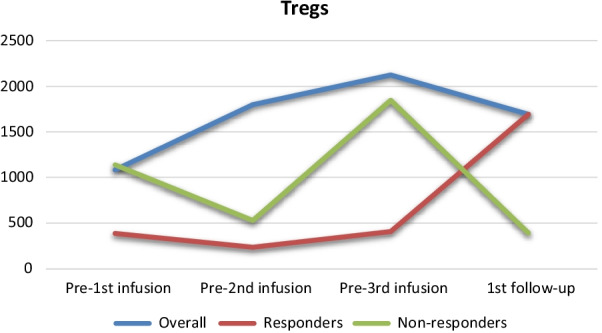
Modification of Tregs in peripheral blood after CB-MSCs therapy

Tregs quantification in peripheral blood was performed in 10/11 patients. The modification of Tregs after CB-MSC infusions are summarized in Fig. 1. Among responders, Treg count (number of cells/10^6^ plated PBMCs) changed from a median of 384 (130–1607) at baseline to a median of 1694 (0–10,679) at the first follow-up (FU1) 2 weeks after the last infusion, with 2 out of 3 patients having an overall increase. Among non-responders, excluding patients with a genetic mutation which were not assessable for response, Tregs decreased from a median of 1138 (245–24,570) at baseline to 388 (0–1693) at FU1. Overall, CB-MSCs therapy was not associated with a predictable increase of circulating Tregs. However, a correlation between Treg induction and disease remission cannot be excluded.

No significant differences were found in cytokine levels between responders and non-responders.

At the last follow-up, among patients who did not achieve remission, three (patients 1, 5 and 8) progressed to ESKD after a median time of 32 months (range: 15–38 months) from diagnosis, and 8 months (range: 6–18 month) from the intervention. All the remaining patients had stable kidney function throughout the observation [30].

## Discussion

To the best of our knowledge, this is the first trial testing CB-MSCs as a potential therapy in children with MDR-INS. All patients had been unsuccessfully treated with steroids, CNIs and at least one additional regimen with either MMF, RTX or OFA, before the enrollment. In this very complex patient population, we were able to prove the safety of CB-MSCs in pediatric patients with MDR-INS, with none of the participants experiencing any grade toxicity related to the therapy. No adverse reaction was observed. While MSCs have been previously used in other clinical settings, no studies have addressed their safety in such a delicate population of children with MDR-INS. Notably, disease remission occurred in 3 out of 9 assessable patients during the follow-up period; these patients were more likely to have a milder disease at the time of therapy, with a partial response to the previous line of therapy.

Different approaches with immunosuppressive drugs have been attempted in children with MDR-INS, but none have shown clear efficacy, although carrying significant toxicities [30]. MMF had disappointing results [30, 31]. Despite some encouraging reports of RTX in patients with SRNS resistant to CNIs [32, 33], other studies, including a randomized controlled trial, failed to confirm these findings [34, 35]. OFA induced remission in a small number of pediatric MDR patients [36, 37], but larger scale clinical trials are needed in order to establish the therapeutic role of OFA in this population.

In view of the dismal prognosis of MDR-INS and in the absence of a definitive treatment, the immunomodulation provided by CB-MSCs could play a role in the disease management, exploiting its innovative mechanism of action.

Our study was not designed to test efficacy as a primary endpoint, neither to exclude spontaneous remission. It is also possible that the ongoing immunosuppressive therapy was responsible, at least in part, for the remission observed in some of the patients. Nevertheless, this experience provides important insights to help in designing further phase II efficacy studies, whenever possible in the context of a randomized clinical trial.

A lower uPr/uCr and better baseline kidney function seem to be predictors of response to CB-MSCs. We can hypothesize that patients with a milder disease, partially responsive to immunosuppressive treatment, are more susceptible to the effect of CB-MSCs. Therefore, CB-MSCs administration could be explored as a timely add-on therapy to immunosuppressive drugs to improve their efficacy and/or reduce their toxicity.

A careful selection of a homogeneous and adequately sized study population will improve the efficacy in future studies. SRNS itself has a wide clinical spectrum with possible different pathogenic mechanisms and we cannot completely rule out that other subjects, in addition to patients 2 and 4, may had a genetic form of NS, secondary to mutations that were not tested.

Also, the dosing of CB-MSCs and infusion regimen chosen for this trial were based upon previous studies in GVHD patients, but it is possible that this regimen is not suitable for patients with MDR-INS. Indeed, there is still lack of consensus regarding the optimal dosing strategy of MSCs [8, 38].

Another important point is the use of representative biomarkers of clinical response. Increased Tregs levels have been reported following infusions of MSCs [39, 40], and at least part of the immunomodulatory effect of MSCs is attributed to the induction of Tregs [41]. Furthermore, remission of proteinuria in INS was reported following induction of Tregs [42, 43]. However, in our cohort of patients, we were not able to demonstrate a change in the number of peripheral Tregs associated with CB-MSC infusions. The effect of concomitant immunosuppressive regimens, as well as the correct timing of sampling after CB-MSC infusion, may partially explain these results. Therefore, the role of Tregs as biomarker of MSCs activity in INS deserves further investigation.

Finally, the role of the recipient has recently gained growing importance in the interpretation of the clinical response to MSCs. Specifically, the level of inflammatory cytokines as well as the immune-mediated MSC lysis are the main recipient-related factors that have been put in relationship with their clinical efficacy.

In particular, the presence of a pro-inflammatory milieu, mainly IFN-gamma [44], is crucial to let MSCs exert their action. Notably, the baseline levels of pro-inflammatory cytokines in the study population were below those registered in subjects of the same age [28], maybe as an effect of the concomitant immunosuppressive therapy. This may have contributed to the low remission rate in our study.

Moreover, the role of immune-mediated MSC destruction in determining their efficacy has recently been clarified. In a GVHD murine model, Galleu et al. demonstrated that MSC apoptosis induced by the recipient’s cytotoxic cells is essential for MSC-mediated immunosuppression [45]. They also reported a correlation between higher in-vivo cytotoxicity and a clinical response in human patients with GVHD [45]. These findings represent a Copernican revolution in the interpretation of MSC mechanism-of-action and overturn the perspective of preserving MSC viability to guarantee their efficacy. In fact, basing on the state-of-the-art at the time of performing our pilot study, CB-MSCs were selected to have a low or absent apoptosis when texted in a mixed lymphocyte reaction with each recipient’s lymphocytes. On the contrary, the low level of CB-MSCs apoptosis may have contributed to the poor response rate in our trial.

This study has some limitations. The relatively small numbers and the single-arm open label design prevent generalizations regarding the efficacy. Moreover, the treatment schedule, the concurrent immunosuppression and the criteria for CB-MSC selection may have affected the results. Nonetheless, this first pilot study provides important information regarding the role of CB-MSCs in the management of pediatric patients with MDR-INS.

## Conclusions

Our study shows that CB-MSCs are safe and may have a potential role as add-on therapy to the immunosuppressive therapy in pediatric patients with MDR-INS as well as in children with other immune-mediated kidney diseases. This study will open new avenues to investigate in a larger cohort which patients could benefit from such therapy, which biomarkers could predict a response, and what are the ideal dosing regimens of MSCs.

## Supplementary Information

**Additional file 1: Table S1.** Additional clinical information of enrolled patients. **Table S2.** CB MSC dose and quality per patient. **Table S3.** CB MSC dose and quality.

## Data Availability

Main data generated or analyzed during this study are included in this published article. Additional data are available from the corresponding author on reasonable request.

## Abbreviations

CB-MSC: Cord blood mesenchymal stromal cells
CKD: Chronic kidney disease
CNIs: Calcineurin inhibitors
CsA: Cyclosporine A
eGFR: Estimated glomerular filtration rate
ESKD: End-stage kidney disease
GMP: Good Manufacturing Practices
GvHD: Graft versus host disease
IFN: Interferon
IL: Interleukin
INS: Idiopathic nephrotic syndrome
MDR-INS: Multi-drug resistant idiopathic nephrotic syndrome
MMF: Mycophenolate Mofetil
MSC: Mesenchymal stromal cells
OFA: Ofatumumab
PBMCs: Peripheral blood mononuclear cells
RTX: Rituximab
SRNS: Steroid-resistant nephrotic syndrome
TAC: Tacrolimus
Th: T helper
TNF: Tumor necrosis factor
Tregs: Regulatory T cells
uPr/uCr: Urinary protein/urinary creatinine ratio
