# Supplementary material for: First clinical application of cord blood mesenchymal stromal cells in children with multi-drug resistant nephrotic syndrome
Source: Stem Cell Res Ther. 2022 Aug 19;13:420. doi: 10.1186/s13287-022-03112-7 (PMC9389735; doi:10.1186/s13287-022-03112-7)
Supplement: Supplementary file 1 — Additional file 1: Table S1. Additional clinical information of enrolled patients. Table S2. CB MSC dose and quality per patient. Table S3. CB MSC dose and quality. [file 13287_2022_3112_MOESM1_ESM.docx]

Supplementary table 1. Additional clinical information of enrolled patients

|  | **SCREENING** | | | **FIRST INFUSION** | | | **SECOND INFUSION** | | | **THIRD INFUSION** | | | **12 Months** | | |
| --- | --- | --- | --- | --- | --- | --- | --- | --- | --- | --- | --- | --- | --- | --- | --- |
| **ID** | **WEIGHT** | **HEIGHT** | **BMI** | **WEIGHT** | **HEIGHT** | **BMI** | **WEIGHT** | **HEIGHT** | **BMI** | **WEIGHT** | **HEIGHT** | **BMI** | **WEIGHT** | **HEIGHT** | **BMI** |
| **01_01** | 63.6 | 156 | 26.13 | 63 | 156 | 25.89 | 63.1 | 156.6 | 25.73 | 63.9 | 156.6 | 26.06 | 57 | 156.6 | 23.24 |
| **01_02** | 55 | 167.6 | 19.58 | 55 | 167.6 | 19.58 | 55.6 | 168 | 19.70 | 56 | 168 | 19.84 | 56 | 171 | 19.15 |
| **01_03** | 45 | 150 | 20.00 | 42.5 | 152.5 | 18.27 | 42.6 | 152.5 | 18.32 | 42.3 | 153.1 | 18.05 | 46 | 161 | 17.75 |
| **01_04** | 65 | 166.8 | 23.36 | 65.6 | 166.8 | 23.58 | 65.3 | 167 | 23.41 | 65.7 | 167 | 23.56 | 67 | 168.2 | 23.68 |
| **01_05** | 24.5 | 126 | 15.43 | 25 | 126 | 15.75 | 25.3 | 126 | 15.94 | 25.5 | 126 | 16.06 | 26 | 126 | 16.38 |
| **01_06** | 18 | 119.3 | 12.65 | 18.4 | 119.6 | 12.86 | 18.3 | 120 | 12.71 | 18.5 | 120 | 12.85 | 20 | 123 | 13.22 |
| **01_07** | 59 | 163.5 | 22.07 | 61.5 | 163.5 | 23.01 | 62 | 163.5 | 23.19 | 61 | 163.5 | 22.82 | 62 | 164 | 23.05 |
| **01_08** | 41.5 | 153 | 17.73 | 41 | 153 | 17.51 | 40.9 | 153 | 17.47 | 41.2 | 153 | 17.60 | 43.6 | 153 | 18.63 |
| **01_10** | 23 | 122 | 15.45 | 25.8 | 123.6 | 16.89 | 26 | 123.6 | 17.02 | 26.5 | 123.6 | 17.35 | 30.5 | 130.4 | 17.94 |
| **01_11** | 50.5 | 168 | 17.89 | 51 | 168 | 18.07 | 52.5 | 168 | 18.60 | 53 | 168 | 18.78 | 55 | 168.6 | 19.35 |
| **01_12** | 23 | 116.7 | 8.27 | 22.5 | 116.7 | 8.09 | 22.2 | 116.7 | 7.96 | 22.2 | 116.7 | 7.96 | 22.8 | 117.3 | 8.06 |

Supplementary table 2. CB MSC dose and quality per patient

| Patient ID | Donor ID | Cell dose (x10^6^) per treatment | Cell dose (x10^6^/kg) per treatment | Total cell dose (x10^6^cells) | Total cell dose (x10^6^cells/kg) | Purity (%) | Contaminants (%) | Viability (%) |
| --- | --- | --- | --- | --- | --- | --- | --- | --- |
| KID's 01_01 | 2 | 70 | 1.1 | 214.0 | 3.3 | 92 | 0.2 | 84.9 |
| KID's 01_02 | 1 | 70 | 1.3 | 210.0 | 3.9 | 98.2^§^ | 0^§^ | 94.6^§^ |
| KID’s 01_03^ | 3 | 70 | 1.5 | 210 | 4.5 | 99.8^ | 1.2^ | 80.7^ |
| KID’s 01_04 | 1 | 90 | 1.4 | 270 | 4.2 | 98.2^§^ | 0^§^ | 94.6^§^ |
| KID’s 01_05 | 1 | 40 | 1.5 | 120 | 4.5 | 98.2^§^ | 0^§^ | 94.60^§^ |
| KID’s 01_06 | 1 | 37 | 2.0 | 111.0 | 6 | 98.2^§^ | 0^§^ | 94.6^§^ |
| KID's 01_07^ | 3 | 120 | 2.0 | 360.0 | 6 | 99.8^ | 1.2^ | 80.7^ |
| KID’s 01_08 | 1 | 70 | 1.7 | 210 | 5.1 | 99.9 | 0 | 88.45 |
| KID's 01_11 | 2 | 70 | 1.4 | 210 | 4.2 | 99.5 | 0.4 | 90.75 |
| KID's 01_12 | 2 | 35 | 1.5 | 105 | 4.5 | 99 | 0.1 | 93.9 |
| KID's 01_10 | 3 | 40 | 1.6 | 120 | 4.8 | 99.8^ | 1.2^ | 80.7^ |
|  |  |  |  |  |  | 99.2 | 0.5 | 95.1 |

§ and ^: products from the same batch. Patients KID’s 012 received cells from two different batches both from the same donor.

Supplementary table 3. CB MSC dose and quality.

|  | Cell dose (x10^6^) per treatment | Cell dose (x10^6^/kg) per treatment | Total cell dose (x10^6^cells) | Total cell dose (x10^6^cells/kg) | Purity (%) | Contaminants (%) | Viability (%) |
| --- | --- | --- | --- | --- | --- | --- | --- |
| Mean | 64.73 | 1.55 | 194.55 | 4.64 | 97.92 | 0.24 | 90.62 |
| SD | 25.90 | 0.27 | 77.79 | 0.82 | 3.33 | 0.21 | 4.13 |
| Median | 70.00 | 1.50 | 210.00 | 4.50 | 99.20 | 0.20 | 90.75 |
| Min | 35.00 | 1.10 | 105.00 | 3.30 | 92.00 | 0.00 | 84.90 |
| Max | 120.00 | 2.00 | 360.00 | 6.00 | 99.90 | 0.50 | 95.10 |
